# Multifunctional Nanocarriers for diagnostics, drug delivery and targeted treatment across blood-brain barrier: perspectives on tracking and neuroimaging

**DOI:** 10.1186/1743-8977-7-3

**Published:** 2010-03-03

**Authors:** Sonu Bhaskar, Furong Tian, Tobias Stoeger, Wolfgang Kreyling, Jesús M de la Fuente, Valeria Grazú, Paul Borm, Giovani Estrada, Vasilis Ntziachristos, Daniel Razansky

**Affiliations:** 1 Instituto Universitario de Nanociencia de Aragón (INA), Universidad de Zaragoza, Zaragoza, Spain; 2 Zaragoza University Hospital-Miguel Servet, and Instituto Aragonés de Ciencias de la Salud (I+CS), Zaragoza, Spain; 3 Comprehensive Pneumology Centre, Institute of Lung Biology and Disease, Helmholtz Zentrum München, Neuherberg, Germany; 4 Centre of Expertise in Life Sciences, Zuyd University, Heerlen, the Netherlands; 5 Institute of Bioinformatics, Helmholtz Zentrum München, Neuherberg, Germany; 6 Institute of Biological and Medical Imaging, Helmholtz Zentrum München, and Technische Universität München, Germany

## Abstract

Nanotechnology has brought a variety of new possibilities into biological discovery and clinical practice. In particular, nano-scaled carriers have revolutionalized drug delivery, allowing for therapeutic agents to be selectively targeted on an organ, tissue and cell specific level, also minimizing exposure of healthy tissue to drugs. In this review we discuss and analyze three issues, which are considered to be at the core of nano-scaled drug delivery systems, namely functionalization of nanocarriers, delivery to target organs and *in vivo *imaging. The latest developments on highly specific conjugation strategies that are used to attach biomolecules to the surface of nanoparticles (NP) are first reviewed. Besides drug carrying capabilities, the functionalization of nanocarriers also facilitate their transport to primary target organs. We highlight the leading advantage of nanocarriers, i.e. their ability to cross the blood-brain barrier (BBB), a tightly packed layer of endothelial cells surrounding the brain that prevents high-molecular weight molecules from entering the brain. The BBB has several transport molecules such as growth factors, insulin and transferrin that can potentially increase the efficiency and kinetics of brain-targeting nanocarriers. Potential treatments for common neurological disorders, such as stroke, tumours and Alzheimer's, are therefore a much sought-after application of nanomedicine. Likewise any other drug delivery system, a number of parameters need to be registered once functionalized NPs are administered, for instance their efficiency in organ-selective targeting, bioaccumulation and excretion. Finally, direct *in vivo *imaging of nanomaterials is an exciting recent field that can provide real-time tracking of those nanocarriers. We review a range of systems suitable for *in vivo *imaging and monitoring of drug delivery, with an emphasis on most recently introduced molecular imaging modalities based on optical and hybrid contrast, such as fluorescent protein tomography and multispectral optoacoustic tomography. Overall, great potential is foreseen for nanocarriers in medical diagnostics, therapeutics and molecular targeting. A proposed roadmap for ongoing and future research directions is therefore discussed in detail with emphasis on the development of novel approaches for functionalization, targeting and imaging of nano-based drug delivery systems, a cutting-edge technology poised to change the ways medicine is administered.

## Introduction

Nanotechnology has brought a new generation of lightweight materials with superior mechanical and electrical properties. Engineered nanoparticles (NPs) are normally embedded in the matrix of other composites to enhance certain characteristics. Biology and medicine, however, usually employ dispersed NPs, for instance as fluorescent biological labels [1-3], drug and gene delivery agents [4,5], bio-detection of pathogens [6], detection of proteins [7], probing of DNA structure [8], tissue engineering [9,10], tumour destruction via heating (hyperthermia) [11], separation and purification of biological molecules and cells [12], magnetic resonance imaging (MRI) contrast enhancement [13] and phagokinetic studies [14]. The ability of the engineered nanoparticles to interact with cells and tissues at a molecular level provides them with a distinct advantage over other polymeric or macromolecular substances.

While the advent of nanotechnology made its first mark on consumer products, until recently, very little was known about their potential medical applications. NPs have long been noticed to pass across the BBB [15], a tightly packed layer of endothelial cells surrounding the brain that prevents high-molecular weight molecules from passing through. This in itself provides a substantial advantage for drug delivery systems across the BBB, which can pave the way for effective treatments of many central nervous system disorders. This feature, however, was not fully exploited till two decades later.

Despite the advances and breakthroughs in nanotechnology-based approaches, their efficacy towards the treatment of neurological disorders, like brain tumour, stroke, Alzheimer's disease, have been largely constrained. As such, keeping in mind the paucity of therapies for such debilitating disorders, advances in the targeting of drugs to the central nervous system (CNS) will be the main stay for the future success and development of nanotechnology-based diagnostics (application of NPs in therapy and diagnostics) in neurology. To this end, efficient delivery of many potentially therapeutic and diagnostic compounds to specific areas of the brain is hindered by the BBB, the blood cerebrospinal fluid barrier (BCSF), or other specialized CNS barriers [16]. As a result, the global market for drugs for the CNS is greatly under-penetrated and would have to grow by over 500% just to be comparable to the global market for cardiovascular drugs [17]. Only a small class of drugs or small molecules with high lipid solubility and low molecular mass of < 400-500 Daltons actually goes across the BBB [18]. For instance, in a recent study of the comprehensive medicinal chemistry (CMC) database [19], over 7,000 drugs were analyzed and only 5% of these drugs affected the CNS, treating primarily depression, schizophrenia, and insomnia. The average molecular mass of the CNS active drug was 357 Daltons. Another similar study found 12% of drugs active upon the CNS, but only 1% of the total numbers of drugs were active in the CNS for diseases other than affective disorders [20]. Modern ageing societies require therefore a broader spectrum of treatments for neurological disorders.

Functionalization of NPs is indeed the first and perhaps foremost step towards nano-scale drug delivery systems. NPs should inherit a number of desirable characteristics from their functionalization. Drug-carrying capabilities are as important as transport, organ targeting and eventual excretion. Affinity of functional groups to tissue specific transport methods is clearly a challenging problem. It is known that some transport molecules such as growth factors, insulin and transferrin can potentially increase the efficiency and kinetics of drugs across a range of tissues.

Once nanomaterials are enhanced with drug-carrying and transport capabilities, *in vivo *imaging markers, such as fluorescent dyes for optical imaging, is the next landmark to achieve. No review on functionalization of nanocarriers is complete without mentioning imaging technologies capable of their effective visualization. Beyond improvements in overall image quality and spatial resolution, imaging modalities have been entrusted with the challenge of capturing dynamic processes involving various biological system components as well as their respective interactions. For example, the ability to resolve and monitor transmigration ability of various types of biomolecules across the BBB *in vivo *is a daunting challenge. In this context, we give a special attention to the most recent developments in the field of fluorescence-based imaging techniques that have become an integral part of modern biological discovery process, especially in the pre-clinical small-animal-based research. Initially, fluorescence imaging was limited to *ex vivo *and *in vitro *applications with an exception of several intravital microscopy and photographic imaging approaches [21-23]. Although helpful in some cases, these methods fall short to the potential of more recent trans-illumination and tomographic techniques that allow non-invasive fluorescence images *in vivo *[24]. Powerful capabilities are found when those techniques are co-registered with precise *in vivo *anatomical views of the brain provided by MRI or X-ray computed tomography (CT). An additional enormous potential lie ahead with the recent advances of high resolution optoacoustic molecular imaging approaches, such as multispectral optoacoustic tomography (MSOT) [25]. All these are expected to facilitate the development of novel imaging-based diagnostic and therapeutic nanoprobes for early diagnosis and therapy of various disorders of the brain following systematic administration. In this review, we highlight some of the ongoing trends in molecular tomographic imaging of live animals and present insights into exploiting targeting of brain tumours for therapeutic and diagnostics purpose.

Next section will discuss the physiology of BBB, which plays an important role in designing novel platforms to enable access to the brain.

## Blood Brain Barrier: A gateway to neurological diseases

Treatment of neurological diseases such as brain tumours, inborn metabolic errors (e.g., lysosomal storage diseases), infectious diseases and aging, is a daunting challenge due to the unique environment of CNS [26,27]. The advancement of pharmacological drug delivery to the brain has been constrained due the existence of protective barriers which restricts the passage of foreign particles into the brain. Therefore, the efficient design of non-invasive nanocarrier systems that can facilitate controlled and targeted drug delivery to the specific regions of the brain is a major challenge in drug development and delivery for the neurological diseases [28,29]. It becomes crucial to understand the structural composition as well as the functions of the factors that regulate permeability of the substances across the BBB. For that reason, we will briefly discuss the main transporters that mediate the transport of substances across the brain.

### Physiology of the Blood Brain Barrier

**Figure 1:**
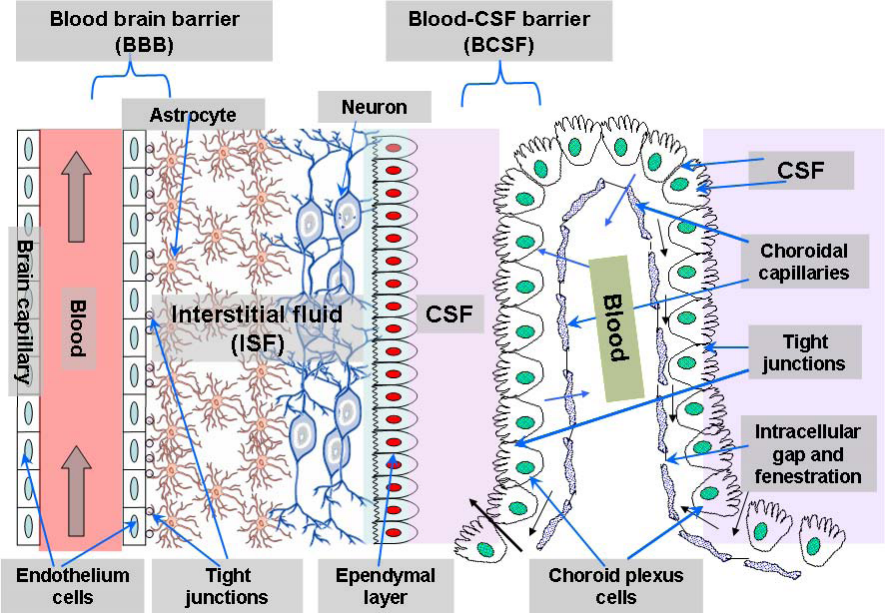
**Overview of the two main barriers in the CNS**. blood-brain barrier and blood cerebrospinal fluid barrier (BCSF). ISF: Interstitial Fluid. CSF: Cerebrospinal fluid. Adapted from [17,18].

Figure 1 gives an overview of the two main immunological barriers, namely BBB and BCSF and their different components. We can see how BBB acts as a neuroprotective shield by protecting the brain from most substances in the blood, supplying brain tissues with nutrients, and filtering harmful compounds from the brain back to the bloodstream [30]. BBB is constituted by the brain endothelial cells which form the anatomical substrate called cerebral microvascular endothelium. It regulates the transport of solutes and other substances including drugs in and out of the brain, leukocyte migration, and maintains the homeostasis of the brain microenvironment, which is crucial for neuronal activity and proper functioning of CNS. The cerebral microvascular endothelium, together with astrocytes, pericytes, neurons, and the extracellular matrix, constitute a "neurovascular unit" that is essential for the health and function of the CNS [31]. The transport of solutes and other substances across BBB is strictly constrained through both physical tight junctions (TJs) and adherents junctions (AJs) and metabolic barriers (enzymes, diverse transport systems) and hence excluding very small, electrically neutral and lipid soluble molecules. Thus, conventional pharmacological drugs or chemotherapeutic agents are unable to pass through the barrier.

**Figure 2:**
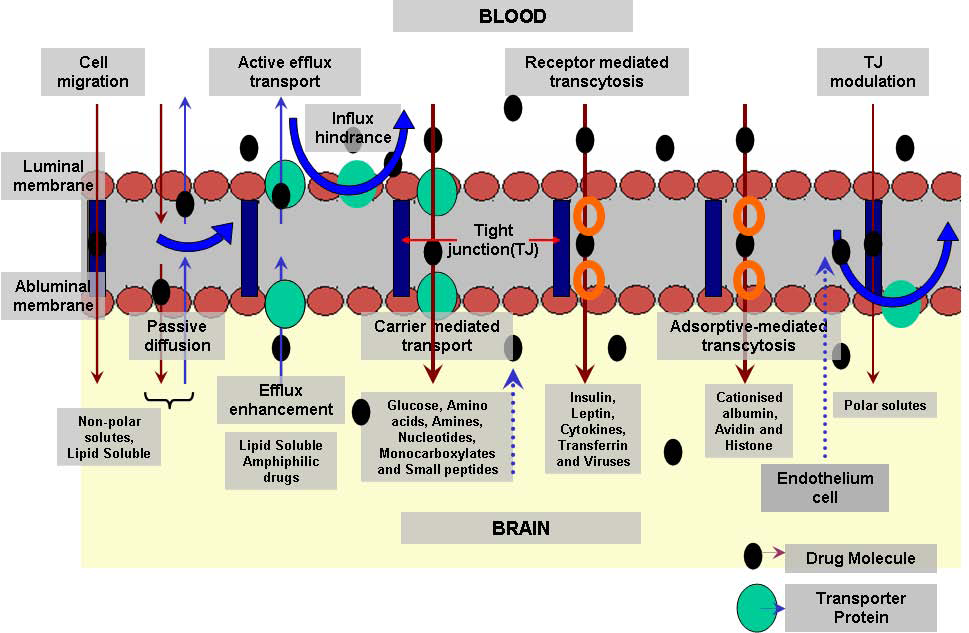
**Potential transport mechanisms across BBB**. Diffusion and active transport as the main transport mechanisms (adapted from [42]).

TJs between endothelial cells of the BBB possess also an intricate complex of transmembrane proteins (junctional adhesion molecule-1 (JAM-1), occludin, and claudins) with cytoplasmic accessory proteins (zonula occludens-1 and -2 (ZO-1 and 2), cingulin, AF-6, and 7H6) and hence acts as physiological and pharmacological barrier, thereby preventing influx of molecules from the bloodstream into the brain. As shown in Figures 1 and 2, BBB is characterized by two membranes, namely luminal and abluminal, facing blood capillary and brain interstitial fluids (ISF), respectively. Another especial feature of BBB is the structural differences that exist between the endothelia of the brain capillaries and endothelia in other capillaries, such as tight junctions between adjacent endothelial cells [31,32], a lack of fenestrations (perforations) and a lack of pinocytotic vesicles [33-38]. Furthermore, in addition to the BBB and BCSF, there exists other CNS barrier shielding the delicate brain tissue from the outer world, but which may play a role in drug transport, such as the blood tumour barrier and the blood retina barrier [39,40], formed of pigment epithelium enclosing the retina, and thereby acting as a barrier interface between the systemic blood vessels of the neighbouring choroid and the retina. Finally targeting of tumour tissue is often constricted by the blood tumour barrier [39].

Moreover, BCSF is the second important feature of the CNS next to the BBB, and is formed by the epithelial cells of the choroid plexus. BCSF controls the penetration of molecules within the interstitial fluid of the brain parenchyma by closely regulating the exchange of molecules between the blood and CSF. Previous reports have demonstrated the following mechanisms of transport pertaining to the choroid plexus: facilitated diffusion (efflux) and active transport into the CSF, as well as active transport (efflux) from CSF to the blood [41-43].

### Role of efflux transporters

The treatment of intractable CNS disorders such as HIV, dementia, epilepsy, CNS-based pain, meningitis and brain cancers depend mainly on the ways to achieve higher drug concentration in the targeted tissues of the brain. The ability of a substance to penetrate the BBB or be transported across BBB is mainly dependent on its physiochemical properties. The total brain exposure, and thus the pharmacological efficacy of a drug or drug candidate, depends on its drug uptake which in turn depends on a combination of factors, including the physical barrier presented by the BBB and the BCSF and the affinity of the substrate for specific transport systems located at both sides of these interfaces [26,27]. The efflux transporters present in the BBB and BCSF, limit brain penetration as well as the intra- and extracellular distribution of a variety of endogenous and exogenous compounds [28].

The efflux transporters role, both as a homeostatic agents against endogenous substances and protective agents against the exogenous substances, have been extensively studied and three classes of transporters have been implicated in the efflux of drugs from the brain: multidrug resistance transporters, monocarboxylic acid transporters, and organic ion transporters [44]. Kabanov et al. [45] have reviewed the inhibition of efflux transporters by Pluronic^® ^block copolymers to enhance the penetration of drugs for CNS delivery. Drug efflux transporters not only cause elimination of the drugs from the brain, but also affects its absorption and tissue distribution [46]. Owing to the growing emphasis on identification and discovery of influx transport proteins (from blood to brain) and efflux transport proteins (from brain to blood) in last years, BBB is now considered to be a dynamic interface that controls the influx and efflux of a wide variety of substances, including endogenous nutrients and exogenous compounds to maintain a favourable environment for the CNS [47].

Deguchi and co-workers demonstrated that the rat organic anion transporter 3 (rOat3) mediated brain-to-blood transport of uremic toxins, as well as that rat organic anion transporting polypeptide (rOatp2) is involved in efflux of 3-carboxy-4-methyl-5-propyl-2-furanpropionate [48]. Sun and co-workers investigated the transport of carbamazepine and drug interactions with cultured rat brain microvascular endothelial cells (rBMEC) as an *in vitro *model of the BBB [49]. They concluded that some specific ABC (ATP-binding cassette, ABC) efflux transporters may be involved in the transport of carbamazepine across the BBB.

The fact that many of the lipophilic drugs show negligible brain uptake can be attributed to the substrates of drug efflux transporters such as the organic anion transporting polypeptides and the BBB active drug efflux transporters of the ATP-binding cassette gene family, e.g. P-glycoprotein (Pgp), multidrug resistance proteins (MRPs) and breast cancer resistance protein (BCRP) [45,48,50-53], that are overexpressed by the endothelial or epithelial cells of these barriers [52]. The combined action of these carrier systems results in rapid efflux of xenobiotics from the CNS and they also account for the cellular localization, specificity, regulation, and potential inhibition at the BBB and BCSF barriers.

Efflux transporters act as a major impediment factor to CNS access by restricting a number of solutes. The future of CNS drug delivery is highly dependent on novel strategies towards modulation of these efflux transporters by designing nanocarriers with tuned affinity for these transporters [45,52,53]. The following section brings a more detailed account of transport mechanisms.

## Mechanisms of transport in and out from the brain

A schematic overview of transport mechanisms across the BBB is shown in Figure 2. There are different mechanisms by which solutes move across membranes in and out of the brain; but nevertheless, all these different mechanisms can be categorized into two basic forms. Firstly, the transport may occur due to diffusion, either simply diffusion or facilitated transport across aqueous channels. The primary bioenergy comes from a concentration gradient across the membranes, between cells (i.e., paracellular) or across cells (i.e., transcellular). This passive diffusion accounts for the transport of solutes through the cell membrane, depending upon size and lipophilicity of the substances [54]. Secondly, active transport is mediated by a carrier such as proteins. The movement may be caused due to the molecular affinity, fluid streams or magnetic fields.

Transports of solutes, drugs and other particles follow different mechanisms as shown in Figure 2 and discussed shortly. Cell migration, in particular that from blood leukocytes like monocytes/macrophages, and T cells circulating through the capillary bed may cross through the BBB driven by chemotaxis, and thereby modifying the functionality of tight junctions [55].

Carrier mediated transport (CMT) or carrier-mediated influx are forms of diffusion which may be passive or active, depending on the context, and involve the unidirectional transport of drugs from the blood to the brain. It is mainly instrumental in the transport of many essential polar molecules, with the help of carrier systems or transporters, such as glucose (GLUT1 glucose transporter), amino acids (the LAT1 large neutral amino acid transporter, the CAT1 cationic amino acid transporter), carboxylic acids (the MCT1 monocarboxylic acid transporter) and nucleosides (the CNT2 nucleoside transporter) into the brain.

Active efflux transport or carrier mediated efflux involve extrusion of drugs from the brain in the presence of efflux transporters such as P-glycoprotein, multidrug resistance protein protein, breast cancer resistance protein and other transporters [56]. In contrast to the carrier mediated transport, the active efflux transport causes the active efflux of drugs from brain back to blood. It acts as a major obstacle in pharmacological drug delivery to the CNS. Interestingly, Banks et al. demonstrated that endogenous peptides like Tyr-Pro-Trp-Gly-NH2, transported from the brain to the blood by peptide transport system-1 (PTS-1), are transported via active efflux [57].

Receptor mediated transport is mainly employed in the transport of macromolecules like peptides and proteins across the BBB by conjugating the substance with ligands such as lactoferrin, transferrin and insulin [58-60]. It is an important transport mechanism of predominant interest in drug delivery. Next, adsorptive mediated transport is a type of endocytosis induced by conjugating the particle to cationised ligands or peptides such as albumin [61,62]. Due to electrostatic interaction with the anionic sites present on the membrane, the cationised ligand conjugated NPs takes the adsorptive mediated transport to enter the brain.

Finally, tight junction (TJ) modulation is caused by the relaxation of junctions, which facilitates selective aqueous diffusion across paracellular junctions in the BBB. Mahajan et al. reported the modulation of tight junction using methamphetamine [63]. Further, they also demonstrated modulation of TJs using Morphine and HIV-1 Tat via the activation of pro-inflammatory cytokines, intracellular Ca2+ release, and activation of myosin light chain kinase [64]. Their studies revealed decreased transendothelial electric resistance and enhanced transendothelial migration across the BBB. Similar observations are known about cocaine on BBB permeability, which indeed worsen HIV dementia. Further studies are needed towards the development of novel anti-HIV-1 therapeutics that target specific TJ proteins, such as ZO-1, JAM-2, Occludin, Claudin-3 and Claudin-5.

Along with the normal physiological delivery methods, a fascinating approach was recently developed using ultrasound-mediated molecular delivery. For instance, see e.g. work by Choi et al. demonstrating deposition of gadolinium through ultrasound-induced blood-brain barrier (BBB) openings in the murine hippocampus [65].

**Figure 3:**
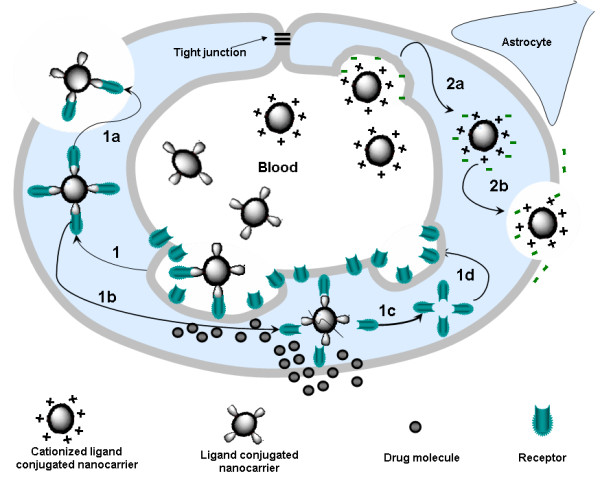
**Mechanisms of drug transport through the BBB using nanocarriers conjugated to receptor-specific ligands and cationized ligands**. (1) Receptor-mediated endocytosis of the nanocarrier; (1a) Exocytosis of the nanocarrier; (1b) Dissociation of the receptor from the ligand-conjugated nanocarrier and acidification of the vesicle leading to the degradation of the nanocarrier and the release of the drug into the brain; (1c and 1d) Recycling of receptors at the luminal cytoplasmic membrane; (2a) Adsorptive-mediated endocytosis of the nanocarrier conjugated to cationized ligands; (2b) Exocytosis of positively charged nanocarriers (adapted from [66]).

One important question in nano drug delivery, however often neglected, is about the fate of the nanocarriers themselves. What happens when nanocarriers (hopefully still carrying the drugs) succeeded in getting access to the central nervous system via BBB? What are the underlying mechanisms that control how these nanocarriers release the therapeutic drugs upon reaching the CNS or the target region? Many of these mechanisms are still not well understood. Dramatic differences can be obtained depending on functionalization, dosages, administration and so on. The main mechanisms involving active targeting are shown in Figure 3. BBB permeability of drugs can be highly increased by active targeting, a non invasive way to transport drugs to target organs using site-specific ligands. Nanocarriers conjugated to ligands capable of recognizing brain capillary endothelial cells and cerebral tumoural cells have emerged as a major breakthrough in CNS drug delivery and Neuro-oncology in particular [66]. The role of endocytosis in targeted brain delivery has been recently reviewed by Bareford et al. and they predicted that by efficient targeting of conjugated nanocarrier systems to the endolysosomal pathway; significant improvement of the drug delivery for the treatment of lysosomal storage diseases, cancer, and Alzheimer's disease can be accomplished. Next, we will discuss about the two main mechanisms of endocytosis mediated transport of nanocarrier systems [67].

### Receptor mediated endocytosis

Receptor mediated endocytosis (RME) or clathrin-dependent endocytosis is a highly specific and energy mediated transport enabling eukaryotic cells to selective uptake macromolecules as specific cargo. For the BBB receptor-specific ligands have also been shown to be very effective to transport endogenous peptides like insulin and transferrin, albumin, and opioid peptides e.g. deltorphins, [D-penicillamine 2,5] enkephalin (DPDPE) and deltorphin II [68-72]. That is why receptor-mediated drug delivery, is also a promising Trojan horse approach for the release of therapeutics into neuronal cells, and tissues. Nanocarriers conjugated to different types of ligands of cell surface receptors expressed on brain endothelial cells, can accumulate and eventually be internalized by cells on the vascular side of the brain through the mechanism of receptor-mediated endocytosis. By direct and indirect conjugation of endogenous and chimeric peptides to nanocarriers or receptors of BBB, significant improvement in drug delivery has been reported [66,73,74]. The desirable fate of targeted receptors after endocytosis can be seen in the in the following way. Upon binding to the receptors, the ligand conjugated nanocarrier gets collected in specialized areas of the plasma membrane known as coated pits. These clathrin coated pits invaginate to form coated vesicles, upon endosomal processing of the vesicle, clathrin and associated proteins dissociate from the vesicle membrane (early endosome), to form new coated pits at the cell surface [70]. The receptor dissociates from the ligand conjugated nanocarrier due to the acidification of the vesicle in the late endosome, and the nanocarrier complex degrades, hence releasing the drug to the cell.

In addition three different mechanisms supporting the ligand conjugated nanocarrier based transport of drugs such as neuropeptides have been proposed: (i) the adsorption of uptake promoting apolipoproteins, (ii) the modulation of tight junctions, and (iii) the inhibition P-glycoprotein, playing a key role in drug resistance [75]. Kreuter et al. suggested that the apolipoproteins B and E may be chiefly involved in the transport of NP-bound drugs into the brain. They concluded that by coating the NPs with polysorbate 80, apolipoproteins B and E get adsorbed onto the NP surface from the blood after injection and thus seem to mimic lipoprotein particles that could be taken up by the brain capillary endothelial cells via receptor-mediated endocytosis [76-78].

After endocytosis, drugs may be released within the endothelium cells and undergo further transportation into the brain by diffusion or through transcytosis [27]. For instance, Liu et al. used chelator-NP system and the chelator-NP system complexed with iron to devise effective therapeutic strategy for Alzheimer's disease which is characterized by dyshomeostasis of metal ions with abnormally high levels of iron in affected areas of the brain [79]. They reported preferential adsorbtion of apolipoprotein E and apolipoprotein A-I in the *in vitro *studies, thereby suggesting the RME transport of chelators and chelator-metal complexes by the NPs across the BBB. Further studies are needed to investigate whether these metal chelators conjugated to NPs can play a role in solubilizing amyloid- [beta] deposits in Alzheimer disease. This can open new pathways to the treatment of neurodegerative diseases and also to study the ways of neural repair using efficiently conjugated nanocarrier system.

Kim et al. recently reported the blocking of low-density lipoprotein receptors (LDLR). Their study is based on brain endothelial cells involving cellular internalization of Poly(methoxy-polyethyleneglycol cyanoacrylate-co-hexa-decyl-cyanoacrylate) (PEG-PHDCA) NPs preincubated with apolipoprotein E. It strengthens the hypothesis of the preponderant role of the LDLR-mediated transport in the endocytosis of PEG-PHDCA NPs [80]. Using protamine-oligonucleotide NPs (proticles) coated with Apolipoprotein A-I (apoA-I), Kratzer et al. observed increased particle uptake and transcytosis in an *in vitro *model of the BBB [81]. These findings were further supplemented by Petri et al. who used Poly(butyl cyanoacrylate) NPs coated with poloxamer 188 (Pluronic^® ^F68) bounded to doxorubicin and reported enhanced anti-tumour effect of doxorubicin against an intracranial glioblastoma in rats [82]. They hypothesized that this may be facilitated by the interaction of apolipoprotein A-I, present on the surface of the NPs, with the scavenger receptor class B, type I, the prime receptor for high density lipoprotein/apoA-I that is expressed on brain capillary endothelial cells (BCEC) [81]. Further research is required to reveal the mechanisms behind the interaction between SR-B1 and apoA-1 and their possible role in enhancing the drug delivery via RME pathway. Moreover, the possibility of more than one mechanism, implicated in the interaction of nanocarrier based drug delivery systems with the brain endothelial cells, cannot be ruled out [83].

In a novel approach, Demeule et al. reported the design of a family of Kunitz domain-derived peptides called Angiopeps as a potential brain drug delivery system. Using a *in vitro *model of the BBB and *in situ *brain perfusion, they demonstrated that these peptides, and in particular Angiopep-2, exhibited higher transcytosis capacity and parenchymal accumulation than other receptors such as transferrin, lactoferrin, and avidin. Furthermore, they suggested that the Angioprep-2 endocytosis may be mediated by the low-density lipoprotein receptor-related protein-1 (LRP1) [84].

### Adsorptive-mediated endocytosis

Adsorptive-mediated endocytosis (AME) is a transport mechanism that has gained significant importance recently, and many new drug delivery technologies focus on AME [61,85]. The underlying principle of AME based transport is the electrostatic interaction between a positively charged substance (e.g. cationized peptide such as albumin) and the negatively charged sites on the brain endothelial cell (BEC) surface (e.g. glycoprotein) [61,86]. Dos Santos et al. studied the nature and distribution of anions on the BEC surface *in vitro *and *in situ *and found that the the predominant anion detected on BEC was heparan sulphate (HS) in comparison to the anionic locations observed in endothelia from aorta and epididymal fat micro-vessels [87].

The hypothesis that phagocytic cells of the innate immune system, mainly neutrophils and monocytes, can be exploited as transporters of drugs to the brain has been studied by Afergan et al. *in vitro*, in rats and rabbits by using negatively-charged nano-sized liposomes with double-radiolabeled 3H (in the membrane) and 14C-serotonin (in the core), and fluorescent markers (membrane and core) [88]. They observed a higher brain uptake of liposomal serotonin, 0.138% ± 0.034 and 0.097% ± 0.011, vs. 0.068% ± 0.02 and 0.057% ± 0.01, 4 h and 24 h after IV administration in rats, serotonin liposomes and in solution, respectively. They concluded that monocytes act as key players for the transport of serotonin liposomes.

Alkaloids like cocaine are well-known stimulants of the central nervous system, and its effect upon the BBB has been studied extensively. Alas, little exploited for drug delivery, it actually relaxes tight junctions and induces leukocyte migration. For instance, Liu et al. reported enhanced BBB permeability and pharmacological activity of the endogenous opioid receptor agonist, endomorphin (EM)-1[68]. A series of EM-1 analogs were tested, e.g. N-terminal cationization, C-terminal chloro-halogenation, and unnatural amino acid (D-Ala, Sar, and D-Pro-Gly) substitutions in position 2. They found that in comparison with EM-1, the four D-Ala-containing tetrapeptides and the chloro-halogenated D-Pro-Gly-containing pentapeptide elicited significant and prolonged central-mediated analgesia upon subcutaneous administration. This fact might be interpreted as more peptides reaching the CNS, thus bringing greater analgesic effect. They also reported that the guanidino- [D-Ala2, p-Cl-Phe4]EM-1 showed 3 times more analgesia than the parent peptide following intra cerebral-ventricular injection.

Adsorptive-mediated transport (AME) based transport has been exploited to facilitate gene delivery into brain tumours. Lu et al., for instance, has incorporated plasmid pORF-hTRAIL (pDNA) into cationic albumin-conjugated PEGylated NPs (CBSA-NP) to evaluate the efficacy of CBSA-NP-hTRAIL as a nonviral vector for gene therapy of gliomas [86]. They observed that 30 minutes after IV administration of CBSA-NP-hTRAIL to BALB/c mice bearing IC C6 gliomas. These NPs co-localized with glycoproteins in brain and tumour microvasculature. And, more importantly, cells accumulated in tumour cells. In addition, they reported apoptosis of brain tumour cells *in vivo *and significantly delayed tumour growth. The above results suggest adsorptive-mediated transport is a very promising route of drug and gene delivery across BBB for CNS disorders. More investigation is required to explore other anionic sites on the BEC surface that can be used to design efficient strategies for delivery using nanocarrier systems through adsorptive-mediated transport. Despite of possessing a lower affinity than RME, AME provides a higher capacity than receptor-mediated endocytosis.

As a field on its own, nano-drug delivery requires proper functionalization, profound knowledge of the range of possible routes to and from the central nervous system, as well as ways to verify whether drugs and nanocarriers reach their final destination. We proceed to review some of the most exciting trends in functionalization, delivery and imaging of nanomaterials.

## NP mediated brain delivery systems

Before starting with the functionalization of NPs, it is important to keep in mind a range of useful properties we wish to have in any drug delivery across the BBB. In this context, owing to their small size, customizable surface, improved solubility, targeted drug delivery and multifunctionality, NPs have emerged as potential drug delivery carriers to tissues throughout the body [89]. Yet passing the BBB is particularly difficult. The proper design of such engineered 'nanocarriers' becomes very important in transversing the impermeable membranes to facilitate drug delivery. At the same time, it is also required to retain the drug stability and ensure that early degradation of drugs from the nanocarriers does not take place.

Therefore, for drugs to be successfully delivered to their target, many factors such as its size, biocompatibility, target specific affinity, avoidance of reticuloendothelial systems, stability in blood, or ability to facilitate controlled drug release need to be considered during manufacture of the NPs. Ideal conditions, or wish-list, of any drug are difficult to meet simultaneously. As for nanocarriers to serve as good candidates for drug delivery across the BBB can be summarized as follows [90,28]:

• particle diameter less than 100 nanometers;

• non-toxic, biodegradable and biocompatible;

• stable in blood (i.e., no opsonisation by proteins);

• BBB-targeted (i.e., use of cell surface, ligands, and receptor mediated endocytosis);

• no activation of neutrophils, non-inflammatory;

• no platelet aggregation;

• avoidance of the reticuloendothelial systems;

• prolonged circulation time;

• scalable and cost effective with regard to manufacturing process;

• amenable to small molecules, peptides, proteins or nucleic acids;

• controlled drug release or should exhibit modulation of drug release profiles.

From materials science perspective, the design of such nanocarriers becomes more complicated when it comes to drug delivery to the brain because of its immunologically privileged characteristics which restricts the entry of most pharmaceutical compounds across the BBB. As such, the applicability of nanotechnology in CNS drug delivery has been grossly limited and this may be attributed to the scarcity of strategies that can allow localized and controlled delivery of drugs across the BBB to the desired site of injury or impairment.

### Functionalization and specificity of NPs

**Figure 4:**
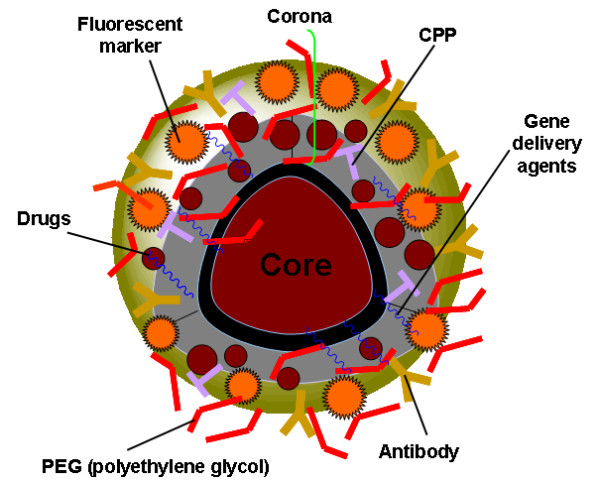
**Schematic representation of a multifunctional NP for diagnostics and drug delivery**. Polyethylene glycol (PEG) copolymers are one of the most popular vehicles for drug delivery. The NPs can be functionalized with suitable fluorescent markers, antibodies against tumoural marker, gene delivery agents and drug molecules coated with a form of PEG. The antibody is using a long linking molecule that allows the antibody to stick to PEG coatings. In contrast, cell penetrating peptides (CPP), employed to trigger rapid cell uptake, are attached using short linkers.

One of the most important challenges in nano-based diagnostics and drug delivery is the functionalization of NPs. Firstly, we need to develop effective conjugation strategies to combine, in a highly controlled way, specific biomolecules to the surface of NPs. Figure 4 shows an example of a PEGylated, multilayer NP (polyethylene glycol, PEG, a popular choice for biocompatible nanocarriers.

**Figure 5:**
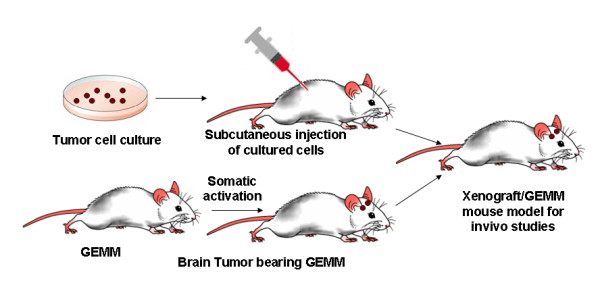
**The Xenograft and genetically engineered mouse model (GEMM)**. In Xenograft mouse models, cancer cells are generally injected subcutaneously into immunodeficient mice. Oncogenes in GEMM are activated and/or tumour-suppressor genes (TSGs) are inactivated somatically.

Some of the most prominent candidate biomolecules are cell penetrating peptides (CPP) such as SynB vectors, penetratin and Tat that facilitate enhanced intracellular delivery [91-95], fluorescent dyes (rhodamine, alexa, Cy5.5), tumoural markers for brain and gene therapeutic agents for genetic therapy such as siRNA [96-101]. Figure 5 show two kinds of mouse tumour models, namely Xenograft and genetically engineered mouse model (GEMM) [102].

Functionalization itself requires a profound knowledge of the target organ and its transport mechanisms. The BBB has several transport molecules that can potentially increase the efficiency and kinetics of nanocarriers towards brains [103], such as, growth factors (e.g. epidermal growth factor [58], vascular endothelial growth factor [104], basic fibroblast growth factor [105], insulin-like growth factors (IGF-I and -II) [106]), biotin-binding proteins (avidin, streptavidin, or neutravidin) [107], insulin [59,69], albumin [108-110], leptin [111,112], lactoferrin [103,113], iron binding protein p97 (melanotransferrin) [114], transferrin [68,115] and Angiopep-2 [84]. Some agents play a pivotal role in enhancing the permeability of nanoprobes through BBB [116-132]. A list of agents/condition and their effects on BBB are summarized in Table 1.

**Table 1:** Effect of different agent(s)/condition(s) on BBB

Agent/condition	Effect on BBB	Reference
Bradykinin, RMP-7	Transient increase of permeability, activates B2 receptors	[116]
VEGF, HIF-1, Deferoxamine,	Increase of permeability and leakage	[117,118]
TNF-alpha, IL-1beta	Moderate increase of permeability	[119]
Tat, Nef, gp120 + IFN-gamma	HIV-1-associated dysfunction	[28-30,120,121]
Low magnetic field (0.15 T)	Moderated increase of permeability	[122,123]
Metalloproteinases	Increase of permeability	[124]
LTC4	Leukotriene-induced permeability	[125,126]
Lipopolysaccharide	Enhance the passage of regulatory proteins	[127,128]
P85	Increase permeability by inhibiting the drug efflux transporter Pgp	[129]
endothelin-1	Dramatic increase of permeability after intracisternal administration	[130]
tPA	Increase permeability via Akt phosphorylation	[131]
PTX	Increased permeability by altering endothelial plasticity and angiogenesis	[132]

Moreover, by altering the surface of polymeric NPs on coating them with different hydrophilic surfactants, such as polysorbate 80 (Tween^® ^80) or other polysorbates with 20 polyoxyethylene units, biocompatible coatings of non-viral gene delivery systems e.g. by poly ethylene glycol (PEG) attachment for siRNA delivery show significant advantage in brain targeting [98].

### NPs for drug delivery: Need of surfactants for BBB transport

Due to its high specificity, NP provides an ideal platform for the transport of drugs across the BBB. The current status of some NP drug delivery platforms and the corresponding encapsulated drugs is summarized in Table 2. All entries refer to *in vivo *experiments.

**Table 2:** NP based drug delivery systems: a list of NP conjugated platforms for delivery across the BBB

NP Platform	Drug (and effects)	References
PBCA NP coated with Polysorbate 80	dalargin (analgesic)	[133,134,137]
PBCA NP coated with Polysorbate 80	doxorubicin (DOX) (anti-tumour antibiotic)	[139,146,147]
PBCA NP coated with Polysorbate 80	kytorphin (analgesic)	[141]
PBCA NP	NMDA receptor antagonist MRZ 2/576 (antagonist)	[140]
PBCA NP coated with Polysorbate 80	tubocurarine (Increased BBB permeability)	[142]
PEG-PHDCA	PrPres Specific Drug in Prion Disease	[143]
PBCA NP coated with Polysorbate 80	tacrine (Anti Alzheimer's Drug)	[144]
PBCA NP coated with Polysorbate 80	rivastigmine (Anti Alzheimer's Drug)	[145]
PBCA NP coated with Polysorbate 80	gemcitabine (anti glioma drug)	[148]
DMAEMA/HEMA (pH sensitive)	paclitaxel	[75]
LDC-polysorbate 80 NPs	diminazene (anti human African trypanosomiasis (HAT))	[153]
DO-FUdR-SLN	5-fluoro-2'-deoxyuridine (FUdR) (Very efficient in brain targeting)	[154]
PBCA NPs, MMA-SPM NPs, and SLNs	stavudine (D4T), delavirdine (DLV), and saquinavir (SQV) (anti HIV agents and enhanced BBB permeability)	[155]
PBCA NPs coated with apolipoprotein B and E	loperamide and dalargin (increased BBB permeability)	[77]

NP-mediated drug transport to the brain strongly depends on the type of surfactant. In Kreuter and Schroeder's labs, 12 different surfactants were coated onto the surface of poly (butylcyanoacrylate) (PBCA) NPs were injected intravenously into mice to evaluate the influence of surfactant on the analgesic effects. The authors reported that only the NPs with polysorbate 20, 40, 60 and 80 coatings produced significant effect and the maximum effect was observed for the PBCA NPs bearing polysorbate 80 coating [133-136]. PBCA NPs coated with surfactants have been successfully used in the delivery of number of drugs across the BBB [137,138], including the peptides (hexapeptide dalargin and the dipeptide kytorphin), anti-tumour antibiotic doxorubicin (DOX), loperamide, the NMDA receptor antagonist MRZ 2/576, and tubocurarine [137-142].

Calvo et al. employed a novel strategy by using PEGylated polycyanoacrylate, NPs (PEG PHDCA) as vector for drug delivery in experimental model of Prion disease [143]. The work showed that the PEG PHDCA particles produced a higher uptake by the spleen and the brain which are both the target tissues of PrPres (an abnormal isoform which is characterized by the accumulation of the host-encoded Prion protein (PrP) in the brain of experimental Prion diseases mice) in comparison to the non-PEGylated NPs. Wilson et al. have used polymeric NPs for drug delivery of anti-Alzheimer's drugs such as tacrine and rivastigmine in the brain of rats [144,145].

Toxicity of conjugated drug-nanocarriers has always been a concern. Gelperina et al. [139] studied the toxicity of DOX bound to polysorbate 80-coated PBCA NPs in healthy rats, and rats with intracranial glioblastoma. No drug-induced mortality occurred with a dose of 3 × 1.5 mg/kg of the DOX NPs formulation on days 2, 5, 8 after tumour implantation. They concluded that the toxicity of DOX bound to NPs was similar, or even lower, than that of free DOX. Other studies aimed at investigating the toxicological profile of doxorubicin bound to NPs employing different dose regimens correlates with the results of this study [146]. Based on the above findings, Pereverzeva et al. hypothesized that the lower toxicity of the nanoparticulate formulation may be due to the altered biodistribution of the drug mediated by the NPs [146]. Wang et al. applied a unique 1% polysorbate-80 coated gemcitabine PBCA NPs (GCTB-PBCA-NPs) to investigate its inhibitory effects in C6 glioma cells *in vitro *and *in vivo *with Sprague Dawley rats [147]. They observed significant increase in the survival time of the rats injected with the formulation compared with the saline control (P < 0.05).

In an interesting approach, You et al. [148] investigated feedback regulated paclitaxel delivery by using pH-Sensitive poly (N, N-dimethylaminoethyl methacrylate (DMAEMA)/2-hydroxyethyl methacrylate (HEMA)) NPs for the triggered release of paclitaxel within a tumour microenvironment. Driven by the fact that the tumours exhibit a lower extracellular pH than normal tissues, the authors found that the paclitaxel release from DMAEMA/HEMA particles can be actively triggered by small, physiological changes in pH (within 0.2-0.6 pH units). It seems to be a promising way to facilitate drug delivery by regulating the tumour microenvironment. Further studies are thus required to explore other factors within tumour microenvironment that can be exploited to enable controlled release of drugs in brain tumours.

### Drug delivery to the brain using Lipid NPs

Liposomes and related lipid structures have long been employed for drug delivery. Lipid NPs, however, are alternative carrier system to traditional colloidal carriers, such as emulsions, liposomes and polymeric particles. These novel carriers have been employed for brain tumour targeting purposes and reviewed in [76]. NPs based on solid lipids come in different types such as "solid lipid NPs" (SLN), "nanostructured lipid carriers" (NLC) and "lipid drug conjugate" (LDC) [149,150]. The breakthrough in advanced conjugation strategies have further led to the emergence of the newer forms of SLN such as polymer-lipid hybrid NPs, nanostructured lipid carriers and long-circulating SLN [151]. Because of its physiochemical characteristics, SLNs have been very successful in comparison to polymeric NPs due to the lower cytotoxicity, higher drug loading capacity, and best production scalability [152].

Back in 2002, Olbrich et al. reported, for the first time, the use of LDC-polysorbate 80 NPs for brain delivery of diminazene to treat second stage human African trypanosomiasis (HAT) [153]. They obtained NPs with a very high drug load of 33% (w/w), despite of the highly water-soluble drug diminazenediaceturate. They concluded that by transforming water-soluble hydrophilic drugs into LDC, NPs got prolonged drug release and targeting to specific sites by intravenous injection. In an another study published shortly afterwards, Wang et al. synthesized 3',5'-dioctanoyl-5-fluoro-2'-deoxyuridine (DO-FUdR) and incorporated it into solid lipid NPs (DO-FUdR-SLN) by a thin-layer ultrasonication technique in order to deliver the drug 5-fluoro-2'-deoxyuridine (FUdR) to the brain. With the average particle size of 76 nm, drug loading of 29.02% and entrapment efficiency of 96.62%, DO-FUdR-SLN proved to be very efficient in *in vivo *brain targeting [154].

More recently, Kuo et al. evaluated the permeability of anti-human immunodeficiency virus (HIV) agents, including stavudine (D4T), delavirdine (DLV), and saquinavir (SQV), across an *in vitro *model of BBB and incorporating them with PBCA NPs, methylmethacrylate-sulfopropylmethacrylate (MMA-SPM) NPs, and SLNs. Their experimental results revealed an enhanced BBB permeability [155]. Their work suggests that the PBCA, MMA-SPM, and SLNs seem promising for the drug delivery and clinical applications in neuro-AIDS treatment.

### Alternatives routes to drug delivery to the brain

No review of drug delivery across BBB is complete without looking at the broad picture of administration routes. A direct drug administration to the brain region, painless and safe, will definitively improve the scenario. However, in the meantime intravenous administration is most popular choice in clinical studies. Some approaches, however, that have been gaining considerable attention, such as oral route, inhalation or intra-tracheal instillation (IT), intranasal drug delivery, convection-enhanced diffusion and intrathecal/intraventricular drug delivery systems in addition to the conventional modes like intravenous administration. Therefore, the administration route of NPs becomes an important criterion of consideration so as to overcome the physiological barriers of the brain and to achieve high drug concentrations therein [58,156-161].

Interestingly, Semmler-Behnke et al. have recently reported the uptake of 1.4 and 18 nm gold NPs in secondary target organs like the brain following intra-tracheal or intravenous application [158]. Moreover, Wang et al. used fluorescence-labeled bovine serum albumin (FBSA) loaded in biodegradable poly(lactic acid-co-glycolic acid) (PLGA) for intraspinal administration of Glial cell line derived neurotrophic factor (GDNF) following contusive spinal cord injury (SCI) and for *in vitro *study [162]. PLGA-FBSA NPs were well absorbed by neurons and glia, indicating that PLGA as a considerable nanovehicle for the delivery of neuroprotective polypeptide into injured spinal cord. Also, local administration of PLGA-GDNF effectively preserved neuronal fibers and led to the hind limb locomotor recovery in rats with SCI. The research opened a new route nanocarrier administration by intraspinal administration.

Two different modes of NP administration in brain tumour mouse models are shown in Figure 5. Once administered the NPs, they reach the site of tumour, and localize it. Once they cross the BBB, the specific ligands or peptides get attached to the specific surface markers expressed on the tumours. Hence, by functionalising NPs with fluorescent dyes could naturally provide *in vivo *imaging of the ongoing biological events during the drug administration as well may act as potential diagnostics labels for early detection and localization of brain tumours.

### *In vivo *pharmacokinetics, biodistribution and safety of NP mediated drug delivery system

Within the requirements of size and charge of effectively deliver drugs via NP carrier systems, there are other challenges that need further attention. Although, much of the work has been focused towards drug delivery with NPs, relatively few studies have focused on the interaction of NPs and their hosts in terms of biodistribution, organ accumulation, degradation and/or toxicology like possible damage of cellular structures or inflammatory foreign body effects. Nanomedicine may find itself at crossroads. It might not be wise to ignore possible adverse effects or toxicity of nanocarriers [4,8,163-165].

Till recently, no pan-European initiative was addressing these concerns. Noteworthy, the European Commission has established the Registration, Evaluation, Authorisation and Restriction of Chemical substances (REACH) which provide safety regulation on substances. Further, Borm et al. have extensively reviewed the potential risks of use of NPs, in a review report commissioned under the European Centre for Ecotoxicology and Toxicology of Chemicals (ECETOC) [166]. We do expect similar commissions worldwide shortly. Nanodrug delivery is seen in its infancy, and works are mostly focusing on particular aspects rather than holistic approaches, e.g. ADME or DMPK. Well-established research protocols like absorption, distribution, metabolism and elimination (ADME), and drug metabolism and pharmacokinetics (DMPK) will surely be part of nanodrug delivery research in the near future [167].

On the distribution side, for instance, Kreyling et al. have extensively studied translocation kinetics and particle size dependency of NPs [77,164,168-170]. In general, smaller NPs show superior translocation kinetics. But, because of their small size might on the other hand cause toxicological effects, see a review by Oberdörster [171]. It is all about a trade-off between drug potency and immunologic surveillance. For example, NPs of size <100 nm need to be used to circumvent macrophage clearance in the lungs [172]. Furthermore, several authors have reported that intrinsic characteristics of NPs, such as aspect ratio and surface area, can be pro-oxidant and pro-inflammatory [31,165,173,174]. Here, the ultra high surface to mass ratio together with new, and often unexpected nanosize specific, material properties related to extreme radii of curvature deserve closer attention [175,176]. Therefore, the use of biopersistent carbon-based, e.g. single or multi-wall carbon nanotubes, or metallic nanocarriers in nano medicine is debatable. These important findings need not discourage genuine efforts in nanodrug delivery, but strength the selection process of materials, shapes and surface treatments [3,4,8,164]. Biodegradable, non-toxic multi-block co-polymers like those based on poly(image-lysine), PEG copolyester and nanogels (e.g. polyethylenimine-PEG) are thus advantageous.

**Figure 6:**
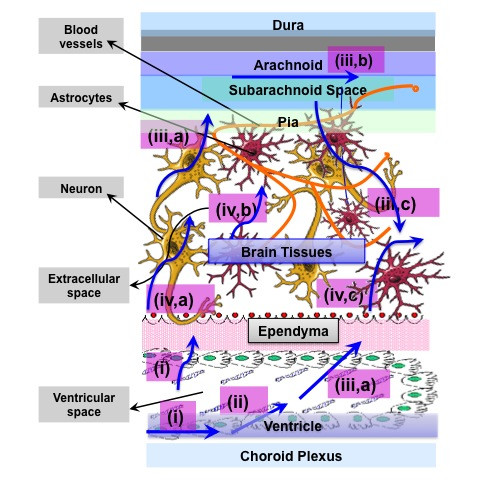
**Fate of drug released from the 'Nanocarrier' systems into the brain**. The main ways drugs and nanocarriers take within the extra cellular space of the brain. (adapted from [177]).

Depending on their functionalization, biodegradable nanocarriers can take a number of paths within tissues. What are the possible trajectories nanocarriers take inside the brain? Pharmacokinetics and excretion are key points that demand an exhaustive research. Figure 6 shows the main ways drugs and nanocarriers take within the extra cellular space of the brain. Following their release, drugs can take different mechanisms and may be transported within (and outside) the brain. One of the mechanisms is their transport by diffusion due to drug concentration gradients as shown in Figure 6 (i); or they may be transported because of the convection due to fluid pressure gradients (ii). Figure 6 (iii, a) shows drug migration into ventricular space via pial or ependymal surface. The drug molecules may also undergo circulation in the sub-arachnoid mater or ventricular spaces (iii, b). Subsequently, it is possible to diffuse back into the brain interstitium (iii, c). The drug molecules may also undergo permeation through the endothelium (iv, a); followed by the circulation in the cerebral blood vessels (iv, b); and eventually may re-enter the brain interstitium by permeation (iv, c) [177].

The exact path, drugs and biodegradable nanocarriers take, depends on many factors and its *in vivo *imaging is perhaps the next milestone for nanodrug delivery, as discussed in the following section..

## Towards development of neurodiagnostic nanoimaging platform

With the advent of multifunctional NPs, the field of brain imaging is encountering a drastic change in the ways one can monitor events at molecular and cellular level as well as to track the development of neurological diseases, cancerous formations etc. One important aspect is development of suitable imaging platforms that can be used to trace these agents *in vivo*. Many of the well-established modalities like positron emission tomography (PET), single photon emission computed tomography (SPECT), MRI, CT, as well as a variety of optical-contrast-based imaging approaches, such as bioluminescence imaging, fluorescence molecular tomography (FMT), and optoacoustic tomography, have gained considerable interest and applicability in neurological research. In the following section, we will focus on some of the commonly used techniques with a special emphasis on the rapidly emerging optical and optoacoustic *in vivo *molecular imaging techniques as well as some trends in multimodality imaging approaches. To better introduce the reader into the modern light-based imaging modalities, we first provide a brief overview of their basic principles of operation and main performance characteristics of the most recent techniques.

### Traditional whole-body imaging modalities

Over the last three decades, X-Ray CT, MRI, and PET have been commonly utilized for visualization of distribution and therapeutic effects of drugs.

X-Ray CT has emerged as a major imaging modality for imaging pharmacokinetics and treatment monitoring, mainly based on indirect tracking of morphological changes. For instance, Rabin et al. reported enhanced *in vivo *imaging of the vasculature, the liver and lymph nodes in mice using a polymer-coated Bi_2_S_3 _NP formulation as an injectable CT imaging agent [178]. Maier-Hauff et al. used CT in order to noninvasively monitor the local drug release in a rabbit radiofrequency (RF) ablation model [179]. Overall, the application of NP based imaging probes to X-ray CT imaging could have a significant impact on health care, owing to the ubiquitous nature of CT in the clinical setting as well as the increasing use and development of micro-CT and hybrid systems that combine PET and SPECT with X-ray CT. Most common CT contrast agents are based on small iodinated molecules, which are indeed effective in absorbing X-rays; but nevertheless, their non-specific distribution, rapid pharmacokinetics and low sensitivity have rather limited their targeting performance.

With the distinct advantage of functional-imaging capabilities as well as better contrast among soft tissues in comparison to the CT, MRI has emerged as a tool in oncological imaging and imaging of the diseased nervous system [180]. Yet, MRI has relatively low sensitivity to exogenous agents, therefore the choice of contrast approaches is of paramount importance in the field of *in vivo *brain imaging. Manganese is gaining importance as T1 contrast neural tracer for MRI. In this role, it was used to study three-dimensional (3-D) connectivity patterns in the rat somatosensory system *in vivo *[181]. To this end, magnetic NPs (MNPs) are of considerable interest as contrast agents for MRI and carriers for drug delivery [182]. Superparamagnetic iron oxide NPs (SPIONs), paramagnetic contrast agent (gadolinium) or perfluorocarbons have already been established as major players in tracking single or clusters of labeled cells within target tissues [183]. Multifunctional nanoplatforms, based on protein cage architectures loaded with imaging agents (fluorophore and MRI contrast agent) onto cells, have also been developed for both diagnostics and targeted treatment [184]. By including gadolinium-loaded liposomes (GDL) with adeno-associated viral vectors (AAV), real time MRI imaging and tracking of convection-enhanced delivery (CED) of viral vectors to the three different regions of non-human primate brain (corona radiata, putamen and thalamus) was achieved [185].

Another non-invasive imaging technology, the positron emission tomography (PET), enables visualization of biodistribution of positron emitter-labelled compounds. PET has certain advantages over CT and MRI, because of its high sensitivity. For instance, Ukrami et al. [186] designed labelled lipid NPs to study *in vivo *distribution of liposome-encapsulated haemoglobin determined by PET. Plotkin et al. [187] employed PET for targeting the intra-tumourally injected magnetic NPs in patients with glioblastoma. Indeed, since its introduction in the late 70's, PET has become a powerful imaging modality with the ability for highly sensitive detection of molecular tracers and is currently utilized in diagnosis, therapy monitoring, and imaging gene expression using diverse reporter genes and probes. However, high costs and other complications associated with PET and SPECT equipment limit their applicability. Moreover, the images acquired by these techniques have poor spatial resolution and hence accurate identification of regions of uptake is difficult to achieve.

In summary, high costs, low sensitivity, and/or low spatial resolution associated with the existing well-accepted clinical imaging modalities promoted the search for new approaches for *in vivo *visualization of brain-targeting nanocarriers, such as methods based on highly sensitive and specific optical contrast.

### Optical imaging

Imaging with light has unique advantages associated with simplicity, low-cost and small size of the equipment. Visible and near-infrared wavelengths offer many probing mechanisms and highly specific contrast approaches not available for other modalities. These can be used for variety of interrogations, from intrinsic functional information on blood oxygenation to molecular sensing [188]. The light radiation is non-ionizing, and therefore reasonable doses can be repeatedly employed without harm to the animal or patient. Optical contrast methods offer the potential to differentiate between soft tissues, due to their distinct light absorption spectra otherwise indistinguishable using other modalities. Also, specific absorption by natural chromophores (such as oxy-haemoglobin) allows functional information to be obtained. The use of extrinsically-administered "switchable" and "tumour-selective" fluorescent optical agents further advances the application possibilities by allowing visualization of otherwise invisible cellular and sub-cellular processes [189-191].

During the last decade, a large number of commercially available fluorescent probes and markers are increasingly being offered, from non-specific fluorescent dyes and fluorescent proteins to targeted or activatable photoproteins and fluorogenic-substrate-sensitive fluorochromes to enable a highly potent field for biological imaging. So far, these contrast mechanisms were proven efficient in a number of clinical and small-animal applications, including probing of tissue hemodynamics [192,193], gene expression profiling [194], detecting protease up-regulation associated with cancer growth and inflammation [195,196] continuous monitoring of the efficacy of anti-cancer treatments and other therapeutic drugs [197]. Since many of the probes are developed to fluoresce in the near-infrared (NIR) optical window, where optical absorption is very low so that light can penetrate deeply, fluorescence imaging has been successfully translated from a microscopy level to whole body small animal imaging and clinics [198,199]. The combination of such probes with optical imaging may yield a unique, highly sensitive technology for *in vivo *and real-time imaging of the expression patterns for various enzymes, which are crucially involved in tumour formation and metastasis. A good example are various breast cancer cell lines that have been identified to over-express specific enzymes such as matrix metalloproteinases [200], which are not over expressed in normal cells.

Despite these advantages, optical imaging is severely limited by scattering: thick tissues diffuse and absorb light and significantly reduce the resolution, penetration capabilities and the overall image fidelity [201]. Even state-of-the-art multiphoton microscopy [202] is usually limited to superficial imaging up to a depth of 0.5-1 mm in most living tissues. Recent efforts to image entire embryos for example required naturally transparent specimen or special chemical treatment to clear them from scattering, which is only suitable for post-mortem imaging. Some other macroscopic photographic approaches like epi-fluorescence suffer from similar light diffusion limitations and therefore have low penetration depth, lack quantification abilities, and overall cannot accurately provide depth and size information [203]. Yet, some diffuse optical tomography (DOT) methods were developed that can provide volumetric images of optical contrast in entire human brain [204] with applications ranging from real-time functional neuro-imaging to the detection of hematomas.

**Figure 7:**
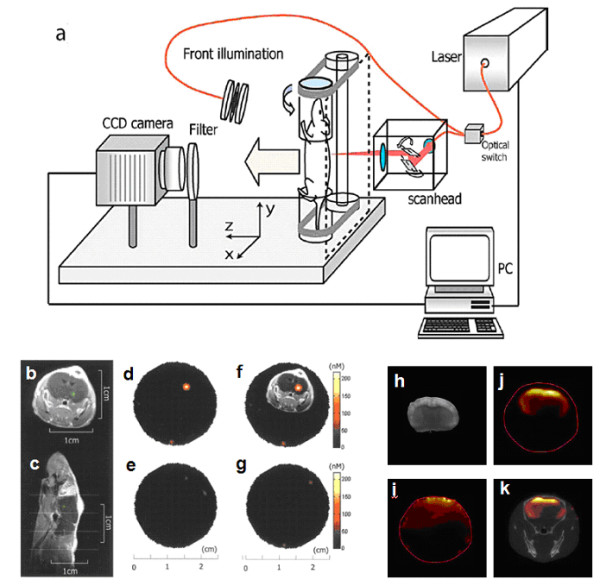
**Summary of different optical diagnostic techniques**. (*a*) Schematic of free-space 360 degree projection FMT imaging system (reprinted with permission from [207]). (b) - (g) In this study, 2 × 10^5 ^cells (9L or HT1080) were stereotactically implanted into unilateral brain hemispheres of nude mice (reprinted with permission from [24]). Animals were then intravenously injected with the cathepsin-B imaging probe (2 nmol Cy 5.5 per animal). (b) and (c) Axial and sagittal MR slices of an animal implanted with a tumour, which is shown in green after gadolinium enhancement. (d), (e), and (f), Consecutive FMT slices obtained from top to bottom from the volume of interest shown on (c) by thin white horizontal lines. (f) Superposition of the MR axial slice passing through the tumour *a *onto the corresponding FMT slice *c *after appropriately translating the MR image to the actual dimensions of the FMT image. (h) - (k) *In vivo *FMT study of Alzheimer's disease progression using a fluorescent oxazine dye to quantify amyloid- [beta] plaques in a transgenic murine model (reprinted with permission from [208]).

It its more advanced form, fluorescence-mediated molecular tomography (FMT) illuminates the sample under investigation at multiple projections and utilizes mathematical models of photon propagation in tissues to reconstruct the underlying imaging contrast in three dimensions, based on distribution of fluorescent molecular probes or fluorescent proteins [195-197,205]. Several different implementations, developed over the past years, have been successfully used to three-dimensionally image bio-distribution of fluorochromes in entire animals, and determine molecular pathways of cancer, neurodegenerative and cardiovascular disease, offering quantitative imaging. Whole-body fluorescence tomography of small animals works optimally in the near-IR region where the lower tissue attenuation allows the penetration of photons over several centimeters [206], but provides low spatial resolution (e.g. on the order of 1 mm in case of whole-body imaging of mice). Figure 7a gives a general schematic of state-of-the-art free-space FMT scanner for *in vivo *tomographic imaging of small animals [207].

FMT systems were so far successfully used in molecular imaging studies of brain disease. In one of the studies [24], using near-infrared fluorescent molecular beacons and inversion techniques that take into account the diffuse nature of photon propagation in tissue, three-dimensional *in vivo *images of protease activity in orthopic gliomas were obtained. In this study, 2 × 10^5 ^cells (9L or HT1080) were stereotactically implanted into unilateral brain hemispheres of nude mice. Animals were then intravenously injected with the cathepsin-B imaging probe (2 nmol Cy 5.5 per animal). The experiments presented the ability of FMT to three-dimensionally and quantitatively resolve fluorochromes in deep tissues and follow their response over time (Figures 7b and 7c).

### Multimodality and hybrid imaging based on optical contrast

Some of optical imaging complications associated with poor spatial resolution and lack of anatomical reference can possibly be mitigated by a marriage between non-invasive optical molecular imaging and other high resolution anatomical imaging modalities such as MRI, or X-Ray CT. The latter combination was recently employed to study the progression of Alzheimer's disease *in vivo *using a fluorescent oxazine dye to quantify amyloid- [beta] plaques in a transgenic murine model [208]. The authors reported very accurate signal localization and correlation of *in vivo *results to *ex vivo *images of excised brain (Figure 7d), thereby emphasizing that FMT is not only a potential tool to study *in vivo *molecular functions, but it can also provide precise mapping of those functions onto high resolution animal anatomy, simultaneously provided by X-Ray CT (Figure 7g). Furthermore, the CT information was used to build a more precise forward model in the FMT image reconstruction process, which also improved spatial resolution and quantification performance of FMT, as can be seen in Figure 7f that was reconstructed using prior structural information (priors) from CT as compared to Figure 7e made without image priors. Another multimodal imaging study was demonstrated by McCann et al. where FMT and MRI were combined to study structure and function of small rodents [209]. Three-dimensional multimodal images were fused to provide a volumetric model of living mouse brains. Interestingly, this approach allows continuous monitoring of tumour morphology, progression and protease activity.

The main challenge for optical imaging of diffuse tissues is degradation of the spatial resolution, which is always exchanged for penetration. As the size of the imaged object grows, imaging resolution quickly deteriorates [210]. It is therefore possible to perform optical tomography, e.g. FMT, through entire mice with high sensitivity, but low resolution of about 1 mm or worse [188,211]. Optoacoustic (or photoacoustic) tomography is an alternative hybrid imaging modality that has recently demonstrated unprecedented high-resolution visualization of optical contrast deep in tissues of small animals [25,212,213]. Optoacoustic imaging relies on detection of ultrasonic signals induced by absorption of pulsed light, thus, high optical absorption contrast can be simultaneously combined with good spatial resolution of ultrasound, not limited by light scattering in tissue. The amplitude of the generated broadband ultrasound waves reflects local optical absorption properties of tissue. Unlike classical optical imaging, the spatial resolution here is not determined nor limited by light diffusion; therefore such performances cannot be achieved by any other optical imaging technology developed so far. Originally, optoacoustic imaging of tissues targeted endogenous tissue contrast, primarily resolving oxy- and deoxy-hemoglobin and different vascular structures. Wang et al. demonstrated high resolution imaging of vascular anatomy in the mouse brain with capability to visualize, with high spatial resolution, functional parameters, e.g. blood oxygenation levels, deep in an intact living mouse brain [212]. Much like the ultrasound, optoacoustics can form images in real time, currently in 2D but potentially also in 3D [214]. In this way, it can be used for real time tracking of dynamic phenomena, such as fast hemodynamic changes [215], bio-distribution of diagnostic agents or pharmacokinetics. However, recently good contrast was also obtained from other biological tissues that do not contain haemoglobin, like fat, bones, and other internal structures [216]. The method was so far used for high-resolution whole-body visualization of several optically diffusive model organisms whose sizes may vary from sub-millimeter up to a centimeter range, e.g. insects, worms, fishes, and small mammals [25,216,217]. However, since optoacoustics was already successfully applied to brain imaging in primates [218] and whole breast imaging in humans [219], selected clinical implementations are also foreseen. Advantageously, spatial resolution in optoacoustics can be kept relatively high (between 20-200 μm) for the entire penetration range of several millimeters to centimeters of tissue.

In addition to offering rich intrinsic tissue contrast, optoacoustic imaging can also be used to visualize exogenous molecular and functional markers. Naturally, almost all materials in nature absorb light therefore can become potential candidates for providing contrast in optoacoustic imaging. For high contrast imaging, of special interest are compounds having high molar extinction (absorption). Several dedicated agents were so far exploited for enhancing contrast in optoacoustics. Gold NPs of various shapes (nanorods, nanocages, nanoshells) [220], were shown to increase optoacoustic signals *in vivo*. Single-walled carbon nanotubes (SWNT) provide an excellent contrast for optoacoustics and, when conjugated with peptides or other specific targeting compounds, can be used as molecular contrast agent [221].

Clearly, many other dedicated contrast agents could potentially be developed for optoacoustic imaging applications. However, additional studies are required to address a variety of efficiency, BBB penetration capabilities, dosing, safety and toxicity concerns associated with those new contrast agents. Instead, many widely adopted optical contrast agents, such as fluorochromes, can be readily used by applying multispectral optoacoustic tomography (MSOT) [25]. It uses pulsed illumination at multiple wavelengths in order to spectrally identify reporter molecules with distinct spectral signatures, such as common fluorochromes or other chromophores within the background tissue absorption. In this way, various additional molecularly-relevant information contained in the optical spectrum can potentially be resolved such as fluorogenic or chromogenic bio-markers associated with gene expression, morphogenesis or decease progression. The method is capable of high resolution 3D visualization of molecular probes, such as common optical molecular probes and fluorescent proteins, located deep in scattering living tissues [25,222]. It can therefore simultaneously deliver anatomical, functional and molecular information with both high resolution and penetration capabilities.

In conclusion, even though optoacoustic imaging methods like MSOT are in their infancy from both technical and application standpoints, it is a rapidly emerging field in the imaging sciences that can overcome major limitations of optical imaging while retaining its contrast and sensitivity advantages [223]. It is therefore expected to drastically expand the capabilities of photonic imaging in the field of *in vivo *imaging of drug delivery markers.

Naturally, every imaging modality comes with its own pros and cons and no method can fulfill the complete range of requirements for every application. Table 3 summarizes the main performance characteristics of different imaging modalities, related to their potential use in real-time tracking of nanocarriers in the brain.

**Table 3:** Performance of different modalities applicable for depth-resolved (volumetric) imaging of the CNS.

Imaging method	Anatomical contrast	Molecular/Functional contrast	Sensitivity to contrast agents	Spatial resolution (*)	Penetration depth	Cost	Safety	Applicability
X-Ray CT	Medium	Poor	μmol (10^-6^)	10-500 μm scalable	Whole-body	Medium	Medium	Pre-clinical/ Clinical
MRI	Good	Medium	nmol (10^-9^)	30-500 μm scalable	Whole-body	High	Good	Pre-clinical/ Clinical
PET/SPECT	Poor	Good	fmol (10^-14^)	1 - 5 mm	Whole-body	High	Medium	Pre-clinical/ Clinical
3D light microscopy	Good	Good	fmol (10^-14^)	0.2 - 10 μm	Superficial (<1 mm)	Medium	Good	Pre-clinical
FMT	Poor	Good	pmol (10^-12^)	1 - 2 mm	~20 mm	Low	Good	Pre-clinical
MSOT microscopy/ tomography	Good	Good	pmol (10^-12^)	5 - 200 μm scalable	~30 mm	Low	Good	Pre-clinical/ Clinical

(*) Spatial resolution usually depends on the overall size of the imaged object/area therefore a range is provided

## Discussion and future perspectives

Drug delivery across the BBB is already one of industry's most sought-after routes. Many ageing disorders and tumours require drugs acting on the central nervous system, and the number of patients looking for efficient treatments is constantly increasing. Longer life expectancy should also match better old-age life [224], however, current therapies fall short of the population's expectations. Anatomic features prevent most drugs to be delivered to the CNS across the BBB. By overcoming the physiological barriers of the brain, achieving higher drug concentration will become indeed feasible, which prompts an intensive search for alternative drug delivery routes.

Multifunctional NPs allow delivering pharmaceutical agents into the brain. We reviewed a range of endogenous molecular pathways represented by growth factors, e.g. insulin and transferrin, which when taken advantage of, can increase the efficiency and kinetics of nanocarriers across the BBB. Multifunctional nanocarriers or their combination with other drugs will drive the search for targeting specific areas in the brain and thus enhance therapies. Nanomedicine has yet to make its mark in clinical studies, and we believe therefore that the accumulated experience in the field has reached its critical mass.

Here, we have reviewed part of this exciting progress and research advances within the context of drug delivery and *in vivo *imaging of multifunctional NPs. Those nanocarriers can indeed be functionalized with drugs as well as fluorescent substances therefore their diagnostics and therapeutic potential is enormous. Imaging of function and molecular activity is at the frontier of current research efforts to detect and study a variety of diseases, such as cancer, in a less invasive way. A range of imaging techniques was reviewed. We described well-established radiological imaging techniques and highlighted the recent developments on optical molecular imaging approaches that exploit intrinsic and exogenous bio-markers for *in vivo *gene expression profiling and visualization of different molecular pathways. Imaging of optical contrast can provide both high sensitivity and specificity because background signals can effectively be suppressed by using smart bio-markers, e.g. enzyme-activated fluorescence probes. A proper combination of optical techniques with conventional techniques like CT and MRI can definitely enhance the ways one can quantitatively monitor structure, function and molecular pathways, key features of neurological diseases. Moreover, recent advances in optoacoustic technologies hold a great promise of overcoming scattering-related limitations of optical imaging, eventually shifting the paradigm of whole-body molecular imaging towards high resolution real-time performance.

For a successful nanomedicine approach, all three elements (functionalization, targeting and imaging) have to be further developed. The interest in BBB has steadily grown in recent years, as can be seen from over 5000 papers now listed in PubMed. From the vast literature we concentrate on the inter-relations between functionalization, targeting and imaging; each of these issues deserving comprehensive reviews on their own. Their proper combination can dramatically enhance spatial and temporal resolution, thereby facilitating a unique way to keep track on disease progression as well as on the histological changes in the target tissues. Nanodrug delivery and multimodal imaging could, in principle, treat and monitor tumour status, thus increasing the patient's likelihood of survival.

The translation of NPs in clinical use for therapeutic and diagnostics applications looks promising amidst the recent developments. The field of nanopharmaceuticals is an emerging area of great medicinal interest [225], which aims at developing novel engineered nanoparticles for pharmaceutical applications and show great promise with varied range of applications such as in vaccination, cell therapy and gene therapy [226]. For instance, nanoparticle based drugs gaining considerable interest in pharmaceutical industry and already in clinical practice are liposomal doxorubicin and albumin conjugate paclitaxel [227,228]. In an ongoing Phase I clinical trial at UCSF, California, paclitaxel albumin-stabilized NP formulation (nab-paclitaxel) is being used in treating advanced cancers such as bladder cancer, brain and CNS tumours, breast cancer, etc [229]. In addition, many other NP based diagnostic and therapeutic agents are in clinical trials [230], and future looks promising for the fast growing field of nano diagnostics. Moreover, further initiatives are required to boost the translation of NP formulations from bench to clinics.

Multifunctional nanocarriers for drug targeting and *in vivo *imaging are mature fields, with bright prospects to bring much-needed treatments for neurodegenerative pathologies. However, from a broader perspective, nanocarriers loaded with multiple diagnostic, therapeutic or targeting molecules can pave the way to successfully deal with a large range of other diseases. Application of multifunctional nanocarriers is one of the main driving forces behind our renewed interest in the BBB. Moreover, it has helped to understand the mechanisms that govern structural and composition changes in response to various natural BBB transporters, undesirable toxins, infective viruses like HIV-1, and potential BBB disrupting molecules. Clinical translation of these findings should be fully exploited as to introduce nano-based medicine, a cutting-edge technology poised to change how medicine is administered.

## Competing interests

The authors declare that they have no competing interests.

## Authors' contributions

SB conceptualized the manuscript and wrote the draft. FT, DR and TS contributed in the draft and concept of the paper. All other co-authors contributed in the expansion and revision process. All authors contributed with their experience in the field of NP functionalization, toxicology, *in vivo *imaging and BBB in the conception and critical review of the manuscript. All authors read and approved the final manuscript.
